# Cochrane Reviews on Deworming and the Right to a Healthy, Worm-Free Life

**DOI:** 10.1371/journal.pntd.0004203

**Published:** 2015-10-22

**Authors:** Nilanthi de Silva, Be-Nazir Ahmed, Martin Casapia, H. J. de Silva, John Gyapong, Mwelecele Malecela, A. Pathmeswaran

**Affiliations:** 1 Department of Parasitology, University of Kelaniya, Ragama, Sri Lanka; 2 Department of Microbiology, National Institute of Preventive and Social Medicine (NIPSOM), Dhaka, Bangladesh; 3 Asociacion Civil Selva Amazónica, Iquitos, Perú; 4 Postgraduate Institute of Medicine, University of Colombo, Colombo, Sri Lanka; 5 University of Ghana, Accra, Ghana; 6 National Institute for Medical Research, Dar es Salaam, Republic of Tanzania; 7 Department of Public Health, University of Kelaniya, Ragama, Sri Lanka; University of Queensland, AUSTRALIA

The Cochrane Library has recently published a 160-page, updated systematic review on deworming drugs for soil-transmitted intestinal worms in children, which concludes, “Treating children known to have worm infection may have some nutritional benefits for the individual. However, in mass treatment of all children in endemic areas, there is now substantial evidence that this does not improve average nutritional status, haemoglobin, cognition, school performance, or survival” [1].

In their review, Taylor-Robinson et al. have categorized trials into those that treated only children known to be infected (*n* = 8) and those that treated all children living in an endemic area (*n* = 37). The authors state that according to their analyses, trials that treated only children known to be infected showed that deworming drugs may increase weight gain (low-quality evidence), but that they do not know if there is an effect on cognitive functioning or physical well-being (very low-quality evidence). They also state that in trials treating all children living in an endemic area, deworming drugs had little or no effect on average weight gain (moderate-quality evidence), haemoglobin (low-quality evidence), or cognition (moderate-quality evidence) [1]. These findings may not support the claims that deworming has important health and developmental benefits beyond the removal of worms, but neither do they justify the claim made by Taylor-Robinson et al. as cited above, that there is “substantial evidence that this does not improve average nutritional status,” etc. Lack of evidence to support effectiveness cannot be considered as evidence of ineffectiveness.

The complexity of host–parasite relationships means that even in communities that are highly endemic for soil-transmitted intestinal worms, only a small proportion of individuals have heavy worm burdens that result in obvious morbidity and disease [2]. However, most control programme managers in endemic countries prefer mass deworming (rather than individual treatment after screening) because the commonly used anthelmintics are not only highly effective in reducing the worm burden [3], they have very few side effects, and the cost of screening faecal samples for evidence of infection is several-fold higher than the cost of treatment, as the authors themselves acknowledge [1]. Also, confining treatment to only children known to be infected is not feasible for another reason: most endemic communities have very limited diagnostic laboratory facilities. In our own experience, where deworming programmes have been introduced in endemic communities, parents and teachers often demand regular treatment with anthelmintics, because their children fare better after treatment.

Thus, almost all studies that examine the impact of deworming in endemic communities must necessarily be conducted within a treatment framework that will inevitably dilute any beneficial effects of deworming. This is firstly because uninfected children cannot be excluded, and secondly because the natural distribution of worm burden in a population usually means that most individuals have light infections, while heavily infected children who suffer the most morbidity (and would therefore benefit most from deworming), constitute only a small proportion of infected individuals. In order to fully evaluate the impact of deworming, morbidity measures must be interpreted in light of infection status and intensity. Taylor-Robinson et al., however, do not make any attempt to do so. In applying the methodology of systematic reviews and concluding that mass deworming of children in endemic areas does not improve average nutritional status, haemoglobin, cognition, school performance, or survival, all the authors of the series of Cochrane Reviews on deworming appear to have ignored the essential fact that their methodology is inappropriate in this context.

The Cochrane Review methodology dictates that only randomized controlled trials that meet very strict criteria should be included [4]. Accordingly, of the 59 records identified in the search for the period of the current update (2012–2015), only ten full text articles have been assessed for eligibility, and only four of these have been included in the final analysis. The four studies that are newly included are clearly identifiable, but the list of studies excluded from the review does not appear to be complete, in that it has only one from the period 2012–2015 [1]. We would also like to point out that this updated review has missed out at least one trial that examined the impact of deworming, published during the period 2012–2015 [5]: it is not listed under studies included in the review, nor under studies excluded from the review, nor under additional references. This omission raises doubts regarding the rigor with which the review has been conducted.

While acknowledging the fact that other Cochrane systematic reviews have been of great value in assessing the effectiveness of more conventional forms of drug therapy, we are of the strong view that the Cochrane Systematic Review methodology is not appropriate for assessing the impact of mass deworming programmes in endemic communities in developing countries.

The constitution of the World Health Organization declares, “The enjoyment of the highest attainable standard of health is one of the fundamental rights of every human being…” [6]. The anthelmintics used for mass deworming have undoubted efficacy in treating soil-transmitted helminth infections. We strongly believe that all children have a right to be free of these infections, and that mass deworming is the most cost-effective way of achieving this goal. In questioning the value of mass deworming by using inappropriate methodology, Taylor-Robinson et al. jeopardize funding for deworming programmes that will benefit hundreds of millions of children in developing countries.
